# Rediscovering a species not seen for a hundred years, *Stathmopodatacita* (Meyrick, 1913) (Lepidoptera, Stathmopodidae), with its unusual fern-spore-feeding life history

**DOI:** 10.3897/BDJ.11.e101468

**Published:** 2023-05-12

**Authors:** Zong-Yu Shen, Yu-Feng Hsu

**Affiliations:** 1 Biodiversity Research Center, Academia Sinica, Taipei, Taiwan Biodiversity Research Center, Academia Sinica Taipei Taiwan; 2 Department of Life Science, National Taiwan Normal University, Taipei, Taiwan Department of Life Science, National Taiwan Normal University Taipei Taiwan; 3 Biodiversity Program, Taiwan International Graduate Program, Academia Sinica and National Taiwan Normal University, Taipei, Taiwan Biodiversity Program, Taiwan International Graduate Program, Academia Sinica and National Taiwan Normal University Taipei Taiwan

**Keywords:** *Pyrossia*, Stathmopodinae, microlepidoptera, larval stage, fern-feeding

## Abstract

**Background:**

Despite being the second largest group of vascular plants, ferns are scarcely reported being fed by insects when compared to angiosperms. Within these fern-feeding insects, lepidopterans are poorly represented and are restricted only to specific groups in this speciose order. The consumers specialising on fern spores are even scarcer in the order, with the majority being consumers of vegetative structures. Amongst the fern-spore-feeding Lepidoptera, Stathmopodidae is the family with the highest species diversity, even with a subfamily, Cyprininae Sinev, 2015, specialising on fern spores. However, fern-spore-feeding habit is not restricted to this subfamily. To understand the evolution of fern-spore-feeding within this family and to increase our knowledge of insect-fern evolution, detailed studies on fern-spore feeding stathmopodids are essential.

**New information:**

The present study rediscovered a rare, fern-spore-feeding, stathmopodid micro-moth, *Stathmopodatacita* (Meyrick, 1913), which has not been formally recorded or identified for more than 100 years. We documented the life history of this species and identified several species of *Pyrrosia* (Polypodiaceae, Platycerioideae) as host for the moth’s larvae. A re-description of the fern-feeding moth is also provided as the original description is obscure in terms of character diagnosis.

## Introduction

The family Stathmopodidae (Lepidoptera, Gelechiidae) is distributed throughout the world with most species reported in Asia and Oceania and the members of this group of microlepidoptera can be recognised by the characteristic rosettes of long and rigid bristles on hind leg segments (Sinev 2015). Just like many minute insects, the diversity of Stathmopodidae is underestimated, lacking comprehensive study. Even though more than 350 species have been described in this family, some species have not been recorded after their original description.

*Stathmopodatacita* (Meyrick, 1913) represents such a case. This mysterious species was firstly described by Edward Meyrick in 1913, based on material from Assam, India. Meyrick also established a new genus *Agrioscelis*, Meyrick (1913), with this species as type species. However, none of the diagnostic characters in Meyrick's original description is diagnostic for species in the genus. Sixty years later, Kasy (1973) transferred this species to *Stathmopoda* after dissecting and examining the genitalia of the specimens collected by Meyrick. Afterwards, this species was not reported or mentioned until 2016, when two unidentified specimens from Japan were tentatively labelled as “*Stathmopoda* sp.5” by Terada (2016), who pointed out that that female genitalia was highly similar to *S.tacita*. However, a taxonomic conclusion could not be reached due to the lack of male specimens.

Here, we present the rediscovery of *S.tacita* and found that they utilise spores and the mesophyll of Polypodiosida, an unusual host for Lepidoptera, as larval diet. Fern feeding is uncommon in Lepidoptera, only occurring in a few groups of moths (Fuentes-Jacques et al. 2022a). Consumption of fern spores is even rarer, with this feeding habit only found in three families: Stathmopodidae, Micropterigidae and Tineidae (Lees and Zilli 2019, Fuentes-Jacques et al. 2022a). Amongst these three fern-spore-feeding families, Stathmopodidae has the highest fern-feeding species diversity. Although the subfamily Stathmopodinae represents the highest species richness in Stathmopodidae, there are only three fern-spore-feeding species known in this subfamily, with most of the remaining fern-feeding species restricted to the subfamily Cyprininae Sinev, 2015 (Terada 2016, Shen and Hsu 2020, Shen et al. 2022, Robinson et al. 2022). The recorded fern-spore-feeding species in *Stathmopoda* (*S.aenea* (Braun, 1918), *S.elyella* (Busck, 1909)) are only known to be distributed in the Nearctic Region (Braun 1918, Needham 1947), with an undescribed *Stathmopoda* species reported by Fuentes-Jacques et al. (2022b) in Mexico. The discovery of the fern-spore-feeding habit in *S.tacita* not only increases the record of the uncommon feeding habit in *Stathmopoda*, but also represents the first record of a fern-spore-feeding species of this genus in the Old World.

## Materials and methods

All adult moths were reared from immature stages - collected from their host plants in the wild. Genitalia slides were prepared following procedures given by Common (1990). Terminology of genitalia follows Klots (1970) and Koster and Sinev (2003) and those of wing patterns follow Koster and Sinev (2003). Specimens will be deposited in the Biodiversity Research Museum, Academia Sinica, Taiwan (BRMAS), National Museum of Natural Science, Taiwan (NMNS), National Taiwan Normal University, Taiwan (NTNU) and genitalia plates will be preserved in National Taiwan Normal University, Taiwan (NTNU).

## Taxon treatments

### Stathmopoda tacita

(Meyrick, 1913)

691EB413-0A8D-5360-B57B-45CB5208B8ED

#### Materials

- **Type status:** Other material. **Occurrence:** individualCount: 3; occurrenceID: C79737BB-4897-5A3B-B79F-835315C120C5; **Taxon:** scientificName: Stathmopodatacita (Meyrick, 1913); higherClassification: Insecta; Lepidoptera; Stathmopodidae; Stathmopodinae; **Location:** continent: East Asia; country: Taiwan; county: Taoyuan; locality: Lalashan; decimalLatitude: 24.71; decimalLongitude: 121.44; **Identification:** identifiedBy: "Zong-Yu Shen"; **Event:** samplingProtocol: Two males, one female emerged 13 April 2018, reared from larvae collected on Pyrrosiasheareri collected on 13 March 2018; **Record Level:** type: "PhysicalObject"; language: "en"; institutionCode: "National Taiwan Normal University” (NTNU); basisOfRecord: "PreservedSpecimen"
- **Type status:** Other material. **Occurrence:** individualCount: 1; occurrenceID: 0EA2F9E4-B4F4-5F49-9388-824A97B1D635; **Taxon:** scientificName: Stathmopodatacita (Meyrick, 1913); higherClassification: Insecta; Lepidoptera; Stathmopodidae; Stathmopodinae; **Location:** continent: East Asia; country: Taiwan; county: Taoyuan; locality: Lalashan; decimalLatitude: 24.71; decimalLongitude: 121.44; **Identification:** identifiedBy: "Zong-Yu Shen"; **Event:** samplingProtocol: One female emerged 13 April 2018, reared from larvae collected on Pyrrosiasheareri collected on 7 April 2018; **Record Level:** type: "PhysicalObject"; language: "en"; institutionCode: "National Taiwan Normal University” (NTNU); basisOfRecord: "PreservedSpecimen"
- **Type status:** Other material. **Occurrence:** individualCount: 1; occurrenceID: 6B0048F0-CD88-5B9B-A9A7-CD8F0C31002B; **Taxon:** scientificName: Stathmopodatacita (Meyrick, 1913); higherClassification: Insecta; Lepidoptera; Stathmopodidae; Stathmopodinae; **Location:** continent: East Asia; country: Taiwan; county: Taoyuan; locality: Lalashan; decimalLatitude: 24.71; decimalLongitude: 121.44; **Identification:** identifiedBy: "Zong-Yu Shen"; **Event:** samplingProtocol: One male emerged 16 May 2018, reared from larvae collected on Pyrrosiasheareri collected on 25 April 2018; **Record Level:** type: "PhysicalObject"; language: "en"; institutionCode: "National Taiwan Normal University” (NTNU); basisOfRecord: "PreservedSpecimen"
- **Type status:** Other material. **Occurrence:** individualCount: 11; occurrenceID: 4820453E-976C-593A-81AB-A97CBCBFF260; **Taxon:** scientificName: Stathmopodatacita (Meyrick, 1913); higherClassification: Insecta; Lepidoptera; Stathmopodidae; Stathmopodinae; **Location:** continent: East Asia; country: Taiwan; county: Nantou; locality: Beidongyanshan; decimalLatitude: 24.07; decimalLongitude: 121.13; **Identification:** identifiedBy: "Zong-Yu Shen"; identificationRemarks: Genitalia slides ZYS-0003; **Event:** eventID: Seven males, four females emerged 13 December 2018－2 January 2019, reared from larvae collected on Pyrrosialingua collected on 13 November 2018; **Record Level:** type: "PhysicalObject"; language: "en"; institutionCode: "National Taiwan Normal University” (NTNU); basisOfRecord: "PreservedSpecimen"
- **Type status:** Other material. **Occurrence:** individualCount: 2; occurrenceID: B646A480-61F1-567B-AED8-BF5727AEBF7F; **Taxon:** scientificName: Stathmopodatacita (Meyrick, 1913); higherClassification: Insecta; Lepidoptera; Stathmopodidae; Stathmopodinae; **Location:** continent: East Asia; country: Taiwan; county: Hualian; locality: Bilv Divine Tree; decimalLatitude: 24.18; decimalLongitude: 121.40; **Identification:** identifiedBy: "Zong-Yu Shen"; **Event:** samplingProtocol: One male, one female emerged 4－17 January 2019, reared from larvae collected on Pyrrosiasheareri collected on 12 December 2018; **Record Level:** type: "PhysicalObject"; language: "en"; institutionCode: "National Museum of Natural Science” (NMNS); basisOfRecord: "PreservedSpecimen"
- **Type status:** Other material. **Occurrence:** individualCount: 2; occurrenceID: 69AF196C-EDF0-5777-92D9-1163912D7ACC; **Taxon:** scientificName: Stathmopodatacita (Meyrick, 1913); higherClassification: Insecta; Lepidoptera; Stathmopodidae; Stathmopodinae; **Location:** continent: East Asia; country: Taiwan; county: Hualian; locality: Bilv Divine Tree; decimalLatitude: 24.18; decimalLongitude: 121.40; **Identification:** identifiedBy: "Zong-Yu Shen"; **Event:** samplingProtocol: One male, one female emerged 1 March 2019, reared from larvae collected on Pyrrosiasheareri collected on 8 January 2019; **Record Level:** type: "PhysicalObject"; language: "en"; institutionCode: "National Museum of Natural Science” (NMNS); basisOfRecord: "PreservedSpecimen"
- **Type status:** Other material. **Occurrence:** individualCount: 1; occurrenceID: BCA7F7DA-8450-5739-BE5F-3210BB4DD43; **Taxon:** scientificName: Stathmopodatacita (Meyrick, 1913); higherClassification: Insecta; Lepidoptera; Stathmopodidae; Stathmopodinae; **Location:** continent: East Asia; country: Taiwan; county: Taichung; locality: Daxueshan; decimalLatitude: 24.24; decimalLongitude: 120.98; **Identification:** identifiedBy: "Zong-Yu Shen"; **Event:** samplingProtocol: One female emerged 2 March 2019, reared from larvae collected on Pyrrosialingua collected on 24 January 2019; **Record Level:** type: "PhysicalObject"; language: "en"; institutionCode: "National Taiwan Normal University” (NTNU); basisOfRecord: "PreservedSpecimen"
- **Type status:** Other material. **Occurrence:** individualCount: 1; occurrenceID: 1C8F2C12-4152-5EFC-9E58-B452EC361576; **Taxon:** scientificName: Stathmopodatacita (Meyrick, 1913); higherClassification: Insecta; Lepidoptera; Stathmopodidae; Stathmopodinae; **Location:** continent: East Asia; country: Taiwan; county: Taoyuan; locality: Lalashan; decimalLatitude: 24.71; decimalLongitude: 121.44; **Identification:** identifiedBy: "Zong-Yu Shen"; **Event:** samplingProtocol: One male emerged 9 April 2019, reared from larvae collected on Pyrrosiasheareri collected on 16 March 2019; **Record Level:** type: "PhysicalObject"; language: "en"; institutionCode: "National Taiwan Normal University” (NTNU); basisOfRecord: "PreservedSpecimen"
- **Type status:** Other material. **Occurrence:** individualCount: 7; occurrenceID: B74DB309-4BE1-508C-957F-8A2F80CFE3A5; **Taxon:** scientificName: Stathmopodatacita (Meyrick, 1913); higherClassification: Insecta; Lepidoptera; Stathmopodidae; Stathmopodinae; **Location:** continent: East Asia; country: Taiwan; county: Hualian; locality: Bilv Divine Tree; decimalLatitude: 24.18; decimalLongitude: 121.40; **Identification:** identifiedBy: "Zong-Yu Shen"; **Event:** samplingProtocol: Three males, four females emerged 14 April 2019, reared from larvae collected on Pyrrosiasheareri collected on 23 March 2019; **Record Level:** type: "PhysicalObject"; language: "en"; institutionCode: "National Museum of Natural Science” (NMNS); basisOfRecord: "PreservedSpecimen"
- **Type status:** Other material. **Occurrence:** individualCount: 2; occurrenceID: C0F12DFC-0AF0-595F-BE44-E5501B23A040; **Taxon:** scientificName: Stathmopodatacita (Meyrick, 1913); higherClassification: Insecta; Lepidoptera; Stathmopodidae; Stathmopodinae; **Location:** continent: East Asia; country: Taiwan; county: Hualian; locality: Guanyuan; decimalLatitude: 24.18; decimalLongitude: 121.34; **Identification:** identifiedBy: "Zong-Yu Shen"; identificationRemarks: Genitalia slides ZYS-0144; **Event:** samplingProtocol: One male, one female emerged 22 April 2019, reared from larvae collected on Pyrrosiaporosa collected on 23 March 2019; **Record Level:** type: "PhysicalObject"; language: "en"; institutionCode: "National Taiwan Normal University” (NTNU); basisOfRecord: "PreservedSpecimen"
- **Type status:** Other material. **Occurrence:** individualCount: 1; occurrenceID: 6C8966A9-6F5B-507F-BB98-DC178EB9C368; **Taxon:** scientificName: Stathmopodatacita (Meyrick, 1913); higherClassification: Insecta; Lepidoptera; Stathmopodidae; Stathmopodinae; **Location:** continent: East Asia; country: Taiwan; county: Hsinchu; locality: Jianshi; decimalLatitude: 24.58; decimalLongitude: 121.30; **Identification:** identifiedBy: "Zong-Yu Shen"; **Event:** samplingProtocol: One male emerged 24 December 2019, reared from larvae collected on Pyrrosialingua collected on 13 October 2019; **Record Level:** type: "PhysicalObject"; language: "en"; institutionCode: "National Museum of Natural Science” (NMNS); basisOfRecord: "PreservedSpecimen"
- **Type status:** Other material. **Occurrence:** individualCount: 2; occurrenceID: 99C35488-1AAD-533B-8B6C-2BCAC556FCC6; **Taxon:** scientificName: Stathmopodatacita (Meyrick, 1913); higherClassification: Insecta; Lepidoptera; Stathmopodidae; Stathmopodinae; **Location:** continent: East Asia; country: Taiwan; county: Yilan; locality: Mingchi; decimalLatitude: 24.65; decimalLongitude: 121.47; **Identification:** identifiedBy: "Zong-Yu Shen"; **Event:** samplingProtocol: Two males emerged 2－13 January 2020, reared from larvae collected on Pyrrosialingua collected on 1 December 2019; **Record Level:** type: "PhysicalObject"; language: "en"; institutionCode: "Biodiversity Research Museum, Academia Sinica” (BRMAS); basisOfRecord: "PreservedSpecimen"
- **Type status:** Other material. **Occurrence:** individualCount: 1; occurrenceID: C8A9E462-9359-562B-9FF1-C4DD52BFEF13; **Taxon:** scientificName: Stathmopodatacita (Meyrick, 1913); higherClassification: Insecta; Lepidoptera; Stathmopodidae; Stathmopodinae; **Location:** continent: East Asia; country: Taiwan; county: Hsinchu; locality: Zhenxibao; decimalLatitude: 24.57; decimalLongitude: 121.30; **Identification:** identifiedBy: "Zong-Yu Shen"; **Event:** samplingProtocol: One female emerged 26 March 2020, reared from larvae collected on Pyrrosialingua collected on 15 March 2020; **Record Level:** type: "PhysicalObject"; language: "en"; institutionCode: "Biodiversity Research Museum, Academia Sinica” (BRMAS); basisOfRecord: "PreservedSpecimen"

#### Description

**Figure 1.**
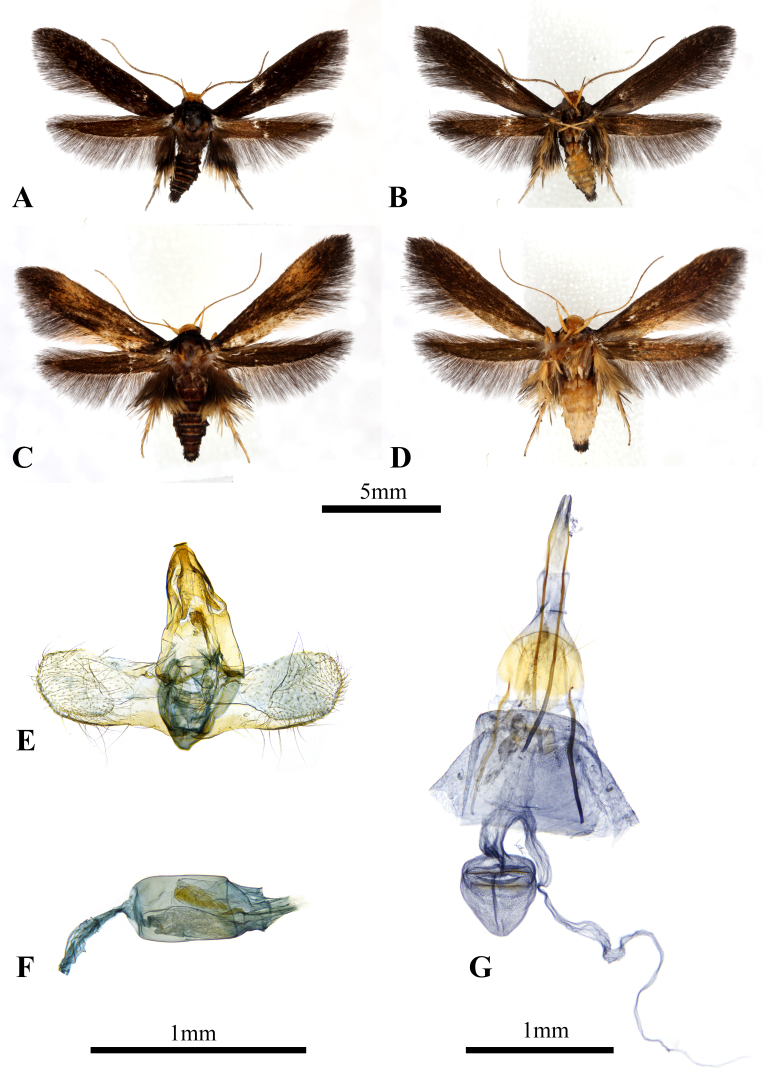
Habitus and genitalia of *Stathmopodatacita* (Meyrick, 1913). **A, B** Male specimen (NMNS). **C, D** Female specimen (NMNS). **E, F** Male genitalia (Gen. Prep. ZYS-0003, NTNU). **G** Female genitalia (Gen. Prep. ZYS-0144, NTNU).

Male (Fig. 1A, B). Forewing length 6.55-7.55 mm (n = 3). Head: frons, vertex, occiput ochre. Antenna with scape rod-shaped, ochre; flagellum ochre, overlaid with fuscous scales on anterior side, with distinct cilia. Labial palps slender, long, strongly upcurved, ochre. Thorax: fuscous, with an ochrous blotch, bilobate, situated at posterior margin of mesothorax; tegula fuscous. Legs: fore- and mid-legs ochre, foretibia and mesotibia overlaid with pale ochrous bristles, mesotibia bearing a pair of pale ochrous spurs at distal joint, with outer spur approximately 1/2 length of inner spur; hind-legs ochre, metatibia overlaid with pale ochrous to fuscous bristles, metatibia bearing two pairs of whitish ochrous spurs at both proximal and distal joints, proximal spurs with outer one approximately 1/2 length of inner one, distal spurs with outer one approximately 3/4 length of inner one. Forewing: ground colour fuscous dorsally; ground colour fuscous ventrally; cilia fuscous. Hind-wing: ground colour dark brown to fuscous, cilia fuscous. Abdomen: dorsally fuscous, pale ochre ventrally, anal tuft present.

Female (Fig. 1C, D). Forewing length 7.61-7.77 mm (n = 3). Similar to male, but lacking cilia on flagellum; forewing with an ochrous blotch, oval, situated at about 1/2 to 9/10 of forewing, ochrous streak present along CuP; anal tuft absent.

Male genitalia (Gen. Prep. ZYS-0003, NTNU, Fig. 1E, F). Uncus mitre-shaped, setae present laterally; gnathos spindle-shaped, distal half laterally tapered and dorsoventrally flattened, tongue-like, with round apex, approximately same length as uncus; tegumen well-developed; valva with round apex, sacculus thicker than costa, with setae ventrally, sclerotised, cucullus approximately 1.25x as long as uncus, with numerous setae on inner surface; saccus approximately 1/4 length as uncus, anellar lobes oval-shaped, with numerous setae; aedeagus stout, approximately 3x as long as uncus; basal sclerotised structure absent; corunutus oblong-shaped, present at about half of aedeagus; apical patch of stimuli present near posterior end of aedeagus, 1/3 length of aedeagus, apically bilobate, narrow, with round apex.

Female genitalia (Gen. Prep. ZYS-0144, NTNU, Fig. 1G). Papillae anales slender, apophyses posterior approximately 2x length as apophyses anterior; ostium bursae oval-shaped, with prominent lamella antevaginalis; corpus bursae with two signa, one on the ventral and one on the dorsal surface of corpus bursae, situated at about 1/5 and 2/5 the length of corpus bursae, bar-shaped; ductus bursae bearing ductus seminalis.

#### Diagnosis

*Stathmopodatacita* is similar to *S.masinissa* Meyrick, 1906 and *S.maritimicola* Terada & Sakamaki, 2011 (see Terada et al. (2011)), but can be distinguished from these two species by the colour and shape of fascia near apex of forewing. The fascia is oval, ochrous in colour in female and vestigial in male in *S.tacita*, whereas the fascia is creamy-white in colour and triangular in both sexes of *S.masinissa*. For *S.maritimicola*, the fascia is triangular, yellow in colour in both sexes and sometimes vestigial in male.

In genitalia, these species can be distinguished by the presence of cervix bursae. Cervix bursae are absent in *S.tacita*, but well-developed in the other two species.

#### Distribution

India (Meyrick 1913) and Taiwan.

#### Biology

**Figure 2.**
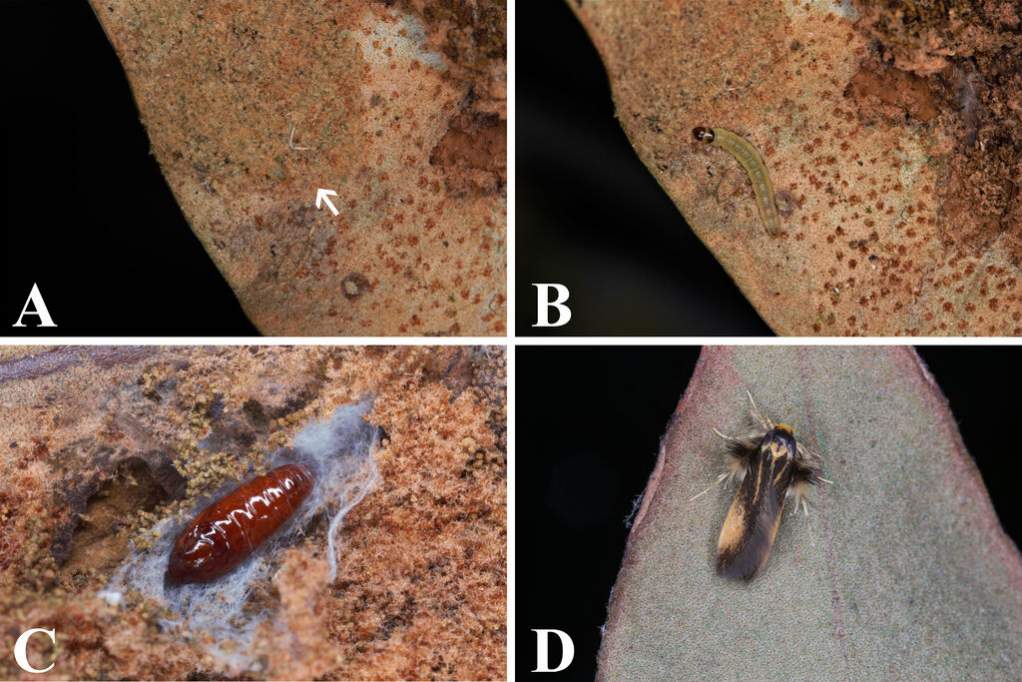
Life history of *Stathmopodatacita* (Meyrick, 1913). **A** The larval shelter (white arrow) on *Pyrrosiasheareri*. **B** The larva of *S.tacita*. **C** The pupa of *S.tacita*. **D** The female adult of *S.tacita*.

Larvae were found in January, March, April, October, November and December. Larvae (Fig. 2B) constructed tunnel-like shelters and live gregariously on the underside of the sporophyll (Fig. 2A). The early instar larvae constructed shelters inside the mid-rib of the host plant and retreated back into the shelters when disturbed. The larvae not only fed on the spores, but also ingested the mesophyll. The pupae were enclosed in loose cocoons within larval shelters. Pupae are oval-shaped, brown and glossy in colour (Fig. 2C). Adult moths (Fig. 2D) emerged about one month after pupation, suggesting that the species is multivoltine.

#### Host plants

*Pyrrosialingua* (Thunb.) Farw., *P.porosa* (Presl) Hovenkamp and *P.sheareri* (Bak.) Ching.

#### Remarks

Kasy (1973) described male genitalia of the species, stating cornutus is absent in the phallus. However, the slightly sclerotised cornutus was actually recognisable in the illustration in his work (see pl. 94), conforming to the specimens from Taiwan. Based on this observation, we consider that a weakly-developed cornutus is present in the male phallus of the species.

## Discussion

Fern-feeding is an uncommon feeding habit in Lepidoptera and is restricted to only a very few groups (Lees and Zilli 2019, Sawamura et al. 2009, Fuentes-Jacques et al. 2022a). Compared to the other fern-feeding lepidopterans, the specialist spore feeders are even rarer. Moreover, spore-feeders are even rarer and largely restricted to the family Stathmopodidae (Fuentes-Jacques et al. 2022a). Understanding how this rare feeding pattern evolved and determining the evolutionary relationships between these species and their fern hosts is of great interest and will require further investigation. To solve these intriguing questions, understanding the systematic relationships of this family is indispensable. We therefore suggest that future studies focus efforts on investigating the biology and systematics of this family. Only with a comprehensive understanding of the intra-family relationships can the study of Stathmopodidae be enhanced.

## Supplementary Material

XML Treatment for Stathmopoda tacita

## References

[B8455976] Braun A. F. (1918). New species of Microlepidoptera. The Canadian Entomologist.

[B8456270] Common I. F.B. (1990). Moths of Australia.

[B8455994] Fuentes-Jacques L. J., Hanson-Snortum P., Hernández-Ortiz Vicente, Díaz-Castelazo Cecilia, Mehltreter K. (2022). A global review and network analysis of phytophagous insect interactions with ferns and lycophytes. Plant Ecology.

[B9559747] Fuentes-Jacques L. J., Hanson-Snortum P., Mehltreter K., Díaz-Castelazo C., Hernández-Ortiz V. (2022). Effects of experimental host-plant switching on the life cycle of a fern spore-feeding micromoth of the genus *Stathmopoda*. Entomologia Experimentalist et Applicata.

[B8456050] Kasy F. (1973). Beitrag zur Kenntnis der familie Stathmopodidae Meyrick, 1913 (Lepidoptera, Gelechioidea). Tijdschrift voor entomologie.

[B8456319] Klots Alexander B. (1970). Lepidoptera. In: Tuxen SL (Ed.). Taxonomist’s glossary of genitalia in insects..

[B8456081] Koster J. C., Sinev S. Y., Huemer P., Karsholt O., Lyneborg L. (2003). Microlepidoptera of Europe.

[B8456094] Lees D. C., Zilli A. (2019). Moths. Their biology, biodiversity and evolution.

[B8456111] Meyrick Edward (1913). Exotic Microlepidoptera.

[B8456119] Needham J. G. (1947). A moth that lives on fern spores (Lepidoptera: Heliodinidae). Proceedings of the Entomological Society of Washington.

[B8456023] Robinson Gaden S., Ackery Phillip R., Kitching Ian J., Beccaloni George W., Hernández Luis M. HOSTs - a Database of the World's Lepidoptera Hostplants. https://www.nhm.ac.uk/our-science/data/hostplants/search/index.dsml.

[B8456128] Sawamura M., Kawakita A., Kato M. (2009). Fern-spore-feeder interaction in temperate forests in Japan: sporing phenology and spore-feeding insect community. American Journal of Bontany.

[B8456137] Shen Z. Y., Hsu Y. F. (2020). The fern-feeding genus *Cuprina* Sinev, 1988 (Lepidoptera, Stathmopodidae), new for Taiwan, with description of two new species. ZooKeys.

[B8456146] Shen Z. Y., Terada T., Hsu Y. F. (2022). The newly recorded fern-spore feeding moths in the genus *Calicotis* Meyrick, 1889 (Lepidoptera: Stathmopodidae) from Taiwan, with notes on life history of three species. Zoological Studies.

[B8456155] Sinev S. Y. (2015). World catalogue of bright-legged moths Lepidoptera, Stathmopodidae).

[B8456189] Terada T., Sakamaki Y., Ohno S. (2011). A new species of the genus *Stathmopoda* (Lepidoptera: Stathmopodidae) closely related to the persimmon pest, *S.masinissa*, from Ryukyu Islands, Japan. Applied Entomology and Zoology.

[B8456181] Terada Takeshi (2016). Stathmopodidae. The Insects of Japan.

